# Evaluation of the efficiency of calcium and vitamin D in treating adults with corticosteroid-induced osteoporosis: A protocol for systematic review and meta-analysis

**DOI:** 10.1097/MD.0000000000027197

**Published:** 2021-10-01

**Authors:** Song-Li Pan, Chen-Cui Li, Hong-Wang Cui, Wen-Xian Wang, Chun-Hong Li

**Affiliations:** a Department of Spine Surgery, the First Affiliated Hospital of Hainan Medical University, 31, Longhua RD, Haikou, Hainan, China; b Department of Clinical Nutrition, the First Affiliated Hospital of Hainan Medical University, 31, Longhua RD, Haikou, Hainan, China; c The First Affiliated Hospital of Hainan Medical University, 31, Longhua RD, Haikou, Hainan, China.

**Keywords:** calcium, efficiency, meta-analysis, osteoporosis, protocol, vitamin D

## Abstract

**Background:**

Administering corticosteroid is an effective therapeutic strategy for treating most inflammatory conditions. However, there is a chance for corticosteroid treatment to adversely affect bones, resulting in corticosteroid-induced osteoporosis, which is a highly prevalent type of secondary osteoporosis. Elevated bone resorption and reduced formation of bone are pathogenesis indicators of corticosteroid-induced osteoporosis. Preventative therapy is recommended for patients initiating steroids. This study aims to evaluate the efficiency of calcium and vitamin D in treating adults diagnosed with osteoporosis caused by corticosteroid therapy.

**Methods:**

Electronic databases will be searched systematically to source studies that have evaluated the efficiency of calcium and vitamin D as a treatment method for adult patients with osteoporosis from corticosteroid therapy. The databases include, PubMed, EMBASE, the Cochrane Library, Scopus, and Web of Science. The timeline of the search will be limited from inception to November 2020. This study will utilize the Cochrane risk of bias tool to assess the quality of the studies reviewed. Moreover, appropriate methods will be chosen to analyze the data. The RevMan 5.3 software is utilized to perform statistical analysis.

**Results:**

This study will provide additional practical and targeted results of evaluating the efficiency of calcium and vitamin D in treating adults with corticosteroid-induced osteoporosis.

**Conclusion:**

The results of this study will provide further evidence about calcium and vitamin D in treating adults with corticosteroid-induced osteoporosis, clinicians and policymakers can make practical use of the results.

**Ethics and dissemination:**

Since this systematic review does not involve any human or animal participants, an ethics approval is not required.

**Systematic review registration:**

Aug 19, 2021. osf.io/zvb38. (https://osf.io/zvb38/).

## 1 Introduction

Osteoporosis is a condition that deteriorates the skeletal system progressively and systemically. The indicators include diminished bone mass and micro-architectural deterioration of bone tissue. Resultantly, the bones are increasingly fragile and highly prone to fractures.^[1]^ The incidence of fractures related to osteoporosis increases sharply with age, from 4% in females aged 50 to 59 to 52% in females aged more than 80 years.^[2]^ Numerous risk factors are related to the development of osteoporosis, such as gender, age, genetic factors, ethnicity, reproductive status, reduced intake of calcium, unhealthy lifestyle, and certain illnesses.^[3]^ Individuals having inflammatory disorders face an elevated risk of developing osteoporosis due to their underlying diseases and frequent intake of corticosteroids. Steroids are widely utilized as a treatment method for bronchial asthma, inflammatory bowel disease, rheumatic disorders, organ transplants, malignant lymphomas, and multiple myeloma. Corticosteroid therapy suppresses the formation of bones and impedes the absorption of Calcium in the intestine, which leads to bone loss and fractures and ultimately to secondary hyperparathyroidism and elevated osteoclastic bone resorption.^[4,5]^ Histomorphometrically, the bone formation rate and trabecular bone volume are reduced.^[6]^ The objectives of treatment include averting bone fractures, sustaining the BMD, and refining physical functions. Calcium and vitamin D are regarded to be at the core of the treatment process in post-menopausal osteoporosis.^[3]^ Several studies investigated how Calcium and Vitamin D affected adult patients with corticosteroid-induced osteoporosis; however, the reported results are not conclusive.^[7–10]^ Thus, this study will be undertaken to evaluate the efficiency of calcium and vitamin D in treating adult patients with corticosteroid-induced osteoporosis.

## 2 Methods

### 2.1 Study registration

This study is funded through a protocol registry in the Open Science Framework (OSF, http://osf.io/). The registration DOI number is 10.17605/OSF.IO/ZVB38. The protocol follows the Preferred Reporting Items for Systematic Reviews and Meta-Analysis Protocol statement guidelines.

### 2.2 Inclusion criteria for study selection

#### 2.2.1 Types of studies

Regardless of the publication status, this review will include all available studies based on the study method of randomized controlled trials (RCTs) that evaluates the efficacy of calcium and vitamin D in treating adults with corticosteroid-induced osteoporosis. The language was restricted to publications in English. Case reports, conferences, reviews, non-RCTs, and non-human studies will be excluded.

#### 2.2.2 Types of participants

All study subjects identified with osteoporosis are included. Patients must take corticosteroids throughout the trial. Patients who had received calcitonin, anti-coagulant drug therapy, and those with any illnesses that might affect bone metabolism will be excluded.

#### 2.2.3 Types of interventions

All study participants will be administered vitamin D with calcium as opposed to vitamin D only, calcium only, or placebo in treating adult patients with corticosteroid-induced osteoporosis.

#### 2.2.4 Types of outcome measures

In this review, the principal outcome measure includes a percentage variation in bone mineral density from the reference. The secondary outcome measures include drop-outs due to side effects, fracture incidence, and 24 hours hydroxyproline excretion.

### 2.3 Data sources and search strategy

#### 2.3.1 Electronic searches

Studies that evaluate the efficiency of calcium and vitamin D in treating adults with corticosteroid-induced osteoporosis will be sourced from PubMed, EMBASE, the Cochrane Library, Scopus, and Web of Science. The time scope is limited from their inception to November 2020. The combination of keywords and free text retrieval is adopted. The following key terms will be used for the search: osteoporosis^∗^, corticosteroid^∗^, steroids^∗^, calcium^∗^, “vitamin D”.

#### 2.3.2 Other resources

Moreover, this study will also refer to the reference lists of the selected studies and search ClinicalTrials.gov (www.ClinicalTrials.gov) and Google scholar to find grey literature.

### 2.4 Data collection and analysis

#### 2.4.1 Selection of studies

**Figure 1:**
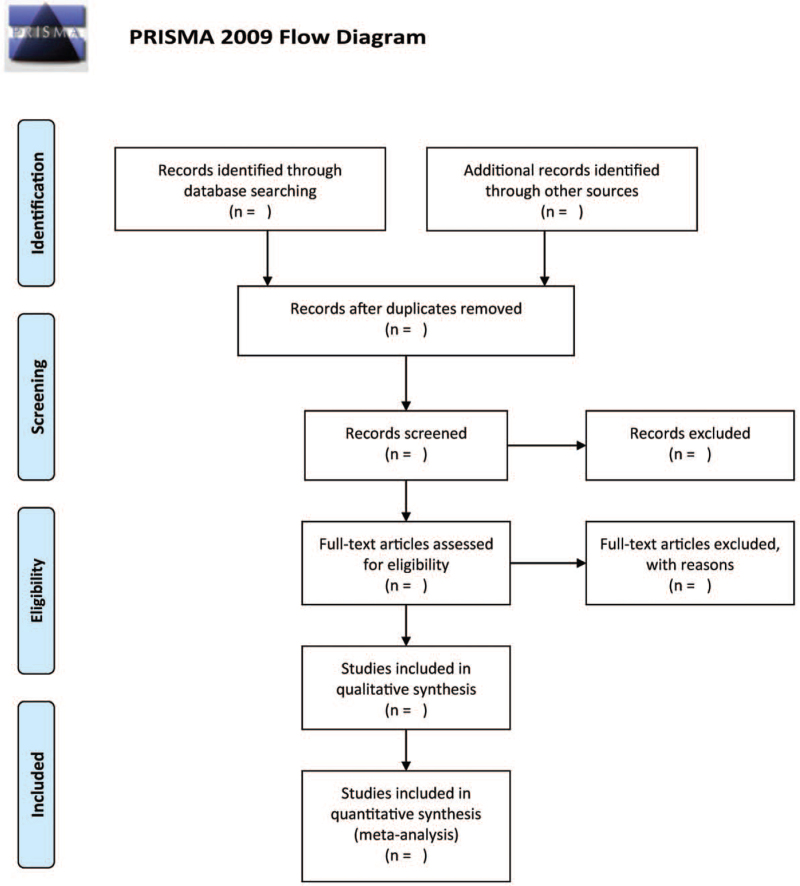
The research flowchart.

Based on the eligibility criteria defined previously, 2 authors will autonomously screen the related studies. Once any duplicates are removed, the titles and abstracts and will be examined. Following a review of the full-text of the preliminary selective papers, the selection of eligible studies is based on our pre-determined inclusion criteria. All disagreements will be resolved through discussion. Figure 1. illustrates the details of the entire selection process.

#### 2.4.2 Data extraction and management

Data extraction will be performed autonomously by 2 authors. The process will use a standard designed data extraction table. The following information will be extracted: first author, publication date, race, gender, disease duration, study method, sample size, intervention details, outcomes, and other associated information. All disagreements will be resolved through discussion.

#### 2.4.3 Risk of bias assessment

The risk of bias in the included studies will be autonomously evaluated by 2 authors, the process will be based on the Cochrane Collaboration's “Risk of bias” tool.^[11]^

#### 2.4.4 Measures of treatment effect

Risk ratios with 95% confidence interval will be used to calculate dichotomous outcomes, and mean difference or standardized mean difference (SMD) with 95% confidence interval to calculate continuous outcomes.

#### 2.4.5 Assessment of heterogeneity

*I*^2^ statistic and the Cochrane's Q test will be used to estimate the heterogeneity among the included studies. Accordingly, an *I*^2^ > 50% or *P* < .1 infers substantial heterogeneity, in which case, the random-effects model will be adopted to merge the results; else, the fixed-effects model is adopted to merge the results.

#### 2.4.6 Assessment of reporting biases

If the number of include studies is more than ten, funnel plots are used to assess the risk of publication bias.^[12]^

#### 2.4.7 Sensitivity analysis

It is planned to carry out a sensitivity analysis to evaluate the robustness of our findings by excluding low-quality studies.

## 3 Discussion

To date, there are no reports for evaluating the efficiency of calcium and vitamin D in treating adults with corticosteroid-induced osteoporosis. Recently, an increasing number of studies have investigated the efficacy of calcium and vitamin D for treating adults with corticosteroid-induced osteoporosis. However, the results of these studies are not conclusive. Therefore, this study is conducted to investigate the efficiency of Calcium and Vitamin D in treating adult patients with corticosteroid-induced osteoporosis. Furthermore, this study also provides helpful recommendations for clinicians and future research on using calcium and vitamin D to treat adult patients with corticosteroid-induced osteoporosis.

## Author contributions

**Conceptualization:** Song-Li Pan.

**Data curation:** Song-Li Pan, Chen-Cui Li, Hong-Wang Cui, Wen-Xian Wang, Chun-Hong Li.

**Formal analysis:** Song-Li Pan, Chen-Cui Li, Hong-Wang Cui, Wen-Xian Wang, Chun-Hong Li.

**Funding acquisition:** Song-Li Pan, Chun-Hong Li.

**Investigation:** Chen-Cui Li, Wen-Xian Wang, Chun-Hong Li.

**Methodology:** Song-Li Pan, Chen-Cui Li, Hong-Wang Cui, Chun-Hong Li.

**Project administration:** Song-Li Pan, Wen-Xian Wang, Chun-Hong Li.

**Resources:** Hong-Wang Cui, Chun-Hong Li.

**Software:** Song-Li Pan, Chen-Cui Li, Hong-Wang Cui, Wen-Xian Wang.

**Supervision:** Wen-Xian Wang, Chun-Hong Li.

**Validation:** Song-Li Pan, Chen-Cui Li, Hong-Wang Cui.

**Visualization:** Song-Li Pan, Chun-Hong Li.

**Writing – original draft:** Song-Li Pan, Chun-Hong Li.

**Writing – review & editing:** Chun-Hong Li.
